# Protein Translation and Signaling in Human Eosinophils

**DOI:** 10.3389/fmed.2017.00150

**Published:** 2017-09-19

**Authors:** Stephane Esnault, Zhong-Jian Shen, James S. Malter

**Affiliations:** ^1^Department of Medicine, Allergy, Pulmonary, and Critical Care Medicine Division, University of Wisconsin-Madison School of Medicine and Public Health, Madison, WI, United States; ^2^Department of Pathology, University of Texas Southwestern Medical Center, Dallas, TX, United States

**Keywords:** eosinophils, protein translation, ribosomal S6 protein, Pin-1, IL-3, intracellular signaling

## Abstract

We have recently reported that, unlike IL-5 and GM-CSF, IL-3 induces increased translation of a subset of mRNAs. In addition, we have demonstrated that Pin1 controls the activity of mRNA binding proteins, leading to enhanced mRNA stability, GM-CSF protein production and prolonged eosinophil (EOS) survival. In this review, discussion will include an overview of cap-dependent protein translation and its regulation by intracellular signaling pathways. We will address the more general process of mRNA post-transcriptional regulation, especially regarding mRNA binding proteins, which are critical effectors of protein translation. Furthermore, we will focus on (1) the roles of IL-3-driven sustained signaling on enhanced protein translation in EOS, (2) the mechanisms regulating mRNA binding proteins activity in EOS, and (3) the potential targeting of IL-3 signaling and the signaling leading to mRNA binding activity changes to identify therapeutic targets to treat EOS-associated diseases.

## Introduction

Control of protein production is critical for the maintenance of cell and tissue homeostasis. Excessive protein production may lead to hypertrophy and an unnecessary use of energy and other resources. However, inadequate protein synthesis antagonizes cell growth, proliferation, adaptation to environmental changes, and the implementation of new cell functions. Overproduction of transcription factors or cytokines contributes to or causes transformation and cancer. Thus, a carefully controlled balance within metabolic constraints but responsive to environmental and signaling cues is essential for optimal cellular function.

Circulating eosinophils (EOS) are differentiated, non-proliferative cells, which become apoptotic within 2–3 days if lacking contact with pro-survival cytokines, such as IL-5, GM-CSF, and IL-3 (1). Therefore, resting EOS have modest needs for new protein production. Protein production is dependent on (1) the level of coding mRNA, which in turn depends on the amount of mRNA transcribed and spliced excluding the amount degraded, and (2) the translation rate of the transcripts, which is governed by ribosomal content, activity, and associated ribosomal and mRNA binding proteins. Extracellular inputs *via* cell surface and intracellular receptors leading to the propagation of intracellular signals control each of these steps [reviewed in Ref. (2)].

**Figure 1:**
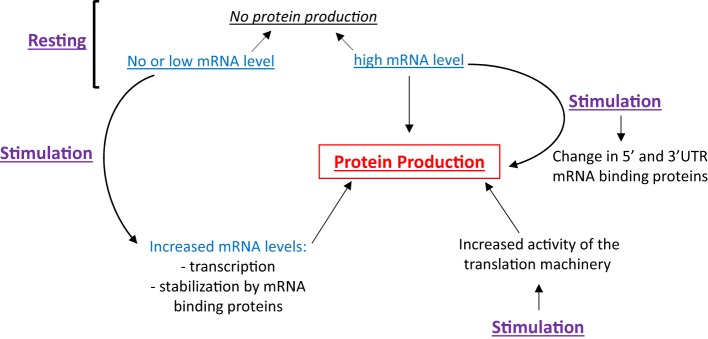
Protein production is a function of cellular stimulation state, mRNA expression level and RNA-binding protein functionality. In resting eosinophils (EOS), protein synthesis can be suppressed irrespective of mRNA content. Cell stimulation can trigger protein production through increased transcription, mRNA stabilization and increased translation, typically regulated by changes in RNABP function.

Eosinophils have the ability to differentially regulate translation. As shown in Figure 1, the presence of high levels of a specific mRNA may or may not lead to protein translation, making inference of protein expression from mRNA quantification tenuous. Cell stimulation can trigger (1) the transcription and translation of mRNA expressed at very low level under basal conditions, (2) the stabilization of mRNA contributing to its accumulation and translation, (3) the translation of mRNA constitutively present but translationally quiescent in resting cells, and (4) an increase in the activity of the machinery, contributing to increased, global protein synthesis. As these topics are far too large to be covered adequately, here we will focus on how changes of both the translation machinery activity and the content of mRNA binding proteins affect the translatability of a subset of mRNA. We will start with an overview of protein translation and its control by intracellular signaling. During this overview, we will use previously published proteomic and phospho-proteomic data from peripheral blood EOS (3) to generalize these known protein translation mechanisms in EOS. Then, we will discuss how changes in mRNA binding proteins and the IL-3-dependent translation of a group of mRNA influence the production of the pro-survival cytokine, GM-CSF, and EOS function, respectively. Finally, the last section, titled “Regulation of translation and potential therapeutic targets,” describes potential molecular drug targets that are implicated in protein translation in EOS in addition to EOS survival and activity. This review may help identify targets that are upstream of GM-CSF and downstream of IL-3 to supplement anti-IL-5 therapies, which despite their efficacy, have not totally controlled eosinophilia and EOS-related pathology. Of note, unless indicated, the observations discussed in this manuscript were obtained using human EOS.

## General Mechanisms Controlling Protein Synthesis

**Figure 2:**
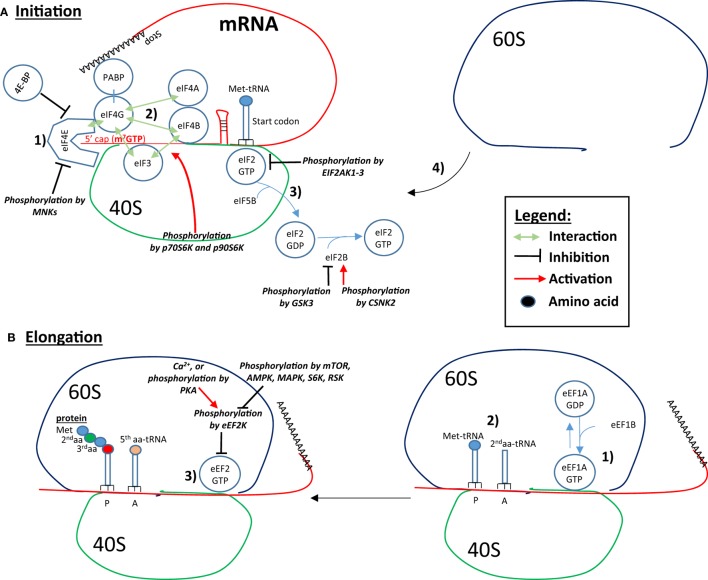
Controls of initiation and elongation of protein translation. **(A)** *Initiation*: (1) eukaryotic translation initiation factor 4E (eIF4E) binds to the 5′-cap structure of the mRNA and eIF4G, which interacts with eIF4A and eIF4B. eIF4G also interacts with the poly-A binding protein (PABP), which circularizes the mRNA and increases the translation rate. (2) eIF4B and eIF4G binds to eIF3, which is associated with the ribosomal 40S subunit, which forms the link between the mRNA, the ribosome and the complex eIF2-GTP/Met-tRNA. In addition, the initiation factors/40S complex scan the 5′UTR until the recognition of the start codon. (3) Protein translation is initiated by the eIF5B-catalyzed eIF2-GTP hydrolysis into GDP, which results in the freeing of the ribosomal 40S from eIF2 and other initiation factors. (4) The recruitment of the 60S ribosomal subunit forms the ribosome by binding to the 40S subunit. eIF4B interaction with eIF3 is increased by p70S6K- and p90S6K-mediated phosphorylation. Binding of eIF4E to eIF4G and to the 5′ cap can be inhibited by 4E-BP and by Mnk-mediated phosphorylation. Phosphorylation of eIF2 by the EIF2AKs inhibits eIF2 recycling. eIF2B is phosphorylated and inhibited by glycogen synthase kinase 3 (GSK3), while phosphorylation of eIF2B by CSNK2 increases its activity toward eIF2 recycling. **(B)** *Elongation*: (1) eEF1A-GTP recruits the second aminoacyl (aa)-tRNA on the A-site. (2) A peptide bond forms between Met and the second aa. (3) eEF2-GTP pushes the mRNA, Met-tRNA is removed from the P-site and is replaced by the next aa-tRNA previously on the A-site. In addition, a third aa-tRNA is placed in the now empty A-site. Eukaryotic elongation factor 2 (eEF2) is inhibited by eEF2K-mediated phosphorylation. EEF2K is inhibited by mTOR, AMP-activated protein kinase (AMPK), mitogen-activated protein kinase (MAPK), S6K, and RSK. Conversely, Ca^2+^ and PKA phosphorylation leads to eEF2K phosphorylation and activation, and inhibition of the elongation.

In eukaryotic cells, initiation, elongation, and termination are the three fundamental steps of protein translation. Some of the main proteins/mRNA interactions involved in the initiation and elongation of translation are shown in Figure 2. During translation, *initiation* begins with the binding of eukaryotic translation initiation factor 4E (eIF4E) to the mRNA 5′-cap. Next, eIF4E binds to eIF4G, which interacts with the other eIF4 proteins, eIF4A and eIF4B. The helicase activity of eIF4A is amplified by eIF4B, and most likely unwinds secondary GC-rich structures of the 5′-UTR, thus facilitating initiation of mRNAs possessing these structures. The interaction of eIF4G with the poly-A binding protein (PABP), which circularizes the mRNA, also increases mRNA translatability. The binding of eIF4B and eIF4G to the 43S preinitiation complex (PIC) *via* eIF3 links the mRNA to the ribosome. The 43S PIC is composed of the ribosomal 40S subunit, eIF3, eIF5 eIF1, eIF1A, and the complex eIF2/Met-tRNA. EIF2 binds Met-tRNA in its GTP-bound state (eIF2-GTP). The complex Met-tRNA/eIF2-GTP along with the initiation factors/40S complex scans the 5′UTR until the start codon (AUG) is recognized by complementarity with the anticodon of Met-tRNA (4). Once the start codon is reached, protein translation becomes initiated by the eIF5B-catalyzed hydrolysis of eIF2-GTP into eIF2-GDP, which frees the ribosomal 40S from eIF2 (5). The release of eIF2-GDP and other initiation factors from the 40S complex is followed by the recruitment of the 60S ribosome subunit. The newly formed 80S ribosomal complex is now ready to start elongation (6). *Elongation* is predominantly controlled by eukaryotic elongation factor 1 (eEF1) and eEF2. Next, eEF1A-GTP recruits the second aminoacyl (aa)-tRNA complementary to the adjacent, C-terminal codon (A-site). After the peptide bound formation between Met and the second aa at the P-site, eEF2-GTP pushes (translocates) the mRNA and allows the third aa-tRNA to become positioned on the third codon at the A-site. Simultaneously, the first Met-tRNA is removed from the P-site and is replaced by the second aa-tRNA previously on the A-site. When the ribosome reaches a stop codon, no complementary tRNA exists to fill the A-site. At that point, the release factor ERF1 (*ETF1*) takes position in the A-site, and along with ERF3A-B (*GSPT1–2*) hydrolyzes the peptide chain (protein) attached to the last t-RNA to *terminate* translation.

### Regulation of Protein Translation

In general, in eukaryotic cells, initiation can be controlled at multiple levels. The eIF4BP proteins (4E-BP) interact with eIF4E, preventing its interaction with eIF4G and, therefore, inhibiting translation initiation (7). 4E-BP are regulated at multiple phosphorylation sites, often by the mammalian target of rapamycin (mTOR), which reduces 4E-BP interactions with eIF4E and enhances translation initiation (8). In addition, the cyclin-dependent kinase 2 (CDK2) phosphorylates 4E-BP on Thr70 leading to its release from eIF4E (9).

Eukaryotic translation initiation factor 4E (eIF4E) can be phosphorylated on Ser209, which decreases its affinity for the 5′-cap structure and, therefore, inhibits translation (10, 11). eIF4E is phosphorylated by the mitogen-activated protein kinase (MAPK) signal-integrating kinases Mnk1 and Mnk2 (*MKNK1* and *2*), which are downstream targets of the MAPK (ERK and p38) (10, 11). Also among the eIF4 family, eIF4B is phosphorylated by p70S6K and p90S6K (RSK) at Ser422, which increases its interaction with eIF3, enhancing translation initiation (12).

When the inactive form of eIF2, eIF2-GDP, leaves the 40S during initiation, it must be recharged with GTP for continuous translation initiation. Then, eIF2-GDP is converted to eIF2-GTP by eIF2B, which is a guanine-nucleotide-exchange factor (GEF). eIF2B is phosphorylated at multiple sites that includes two residues phosphorylated by casein kinase 2 (CSNK2A1) that are required for eIF2B/eIF2 interactions, eIF2 recycling and translation initiation (13). eIF2 is phosphorylated at Ser51 by as many as four kinases, all of which inhibit the eIF2–eIF2B interaction, demonstrating a critical role in protein synthesis (14).

The delivery of aa-tRNA required for *elongation* is driven by the hydrolysis of eEF1A-GTP to eEF1A-GDP. Thus, the GEF eEF1B acts on eEF1A-GDP as eIF2B does on eIF2-GDP. eEF1 is also targeted by a variety of kinases, including PKC, CSNK2, and cyclin-dependent kinase 1 (CDK1), but the role of the phosphorylation states of these elongation factors remains uncertain (15).

Phosphorylation by eEF2 kinase on Thr56 impairs eEF2’s ability to bind to the 40S subunit of the ribosome (16). Thr56 phosphorylation is enhanced if Ser595 is previously phosphorylated (17). The eEF2 kinase activity is calcium/calmodulin-dependent. Its activation after Ca^2+^ flux leads to the attenuation of elongation. Of note, increased eEF2 kinase activity may provide mRNA with poor translation initiation efficiency a greater chance of being synthesized (18). eEF2 kinase is itself regulated at multiple phosphorylation sites, typically by the mammalian target of rapamycin complex 1 (mTORC1) that reduces its activity (19). AMP-activated protein kinase (AMPK) and the MAPK can also phosphorylate eEF2 kinase leading to translation enhancement (18). Conversely, cAMP/PKA signaling pathway phosphorylates Ser500 (20), rending eEF2 activity independent of Ca^2+^ ions and activating the kinase. Figure 2 summarizes these different signaling events and control points.

### General Translation in EOS

Recently, using two-dimensional liquid chromatography coupled with high-resolution mass spectrometry, 6,813 proteins were identified in unstimulated human blood EOS ((3), and *see article by Mosher* et al., *in this issue for more details*). In addition, 4,802 site-specific phosphorylation events were simultaneously identified (3). Furthermore, using RNA-Seq, ~7,981 protein-coding genes expressed by unstimulated human blood EOS were identified (21). The cellular content (mRNA and protein) and phosphorylation state of the main proteins involved in the initiation, elongation, and termination of protein translation have been extracted from the published proteome and transcriptome (shown in Table 1). Notably, Table 1 shows examples of the disconnections between mRNA and protein levels, which suggests that production of certain proteins is tightly regulated at the translational level in EOS (i.e., EIF4G2, ETF1, etc.). For instance, while ratio of protein to mRNA expression generally reached ~1,000 and above, ratios for EIF4G2 and ETF1 were only 107 and 160, respectively (Table 1), suggesting marginal translation for these two transcripts in resting EOS. With the possible exception of the inhibitor of elongation, eEF2K, resting blood EOS possess all the essential proteins involved in protein translation. However, the identification of eEF2 phosphorylation on Thr56 (Table 1) suggests the existence of eEF2K activity, preventing the eEF2/40S interaction and blockade of translation elongation (16). In addition, the lack of phosphorylation of eIF2B (Table 1) suggests a possible lack of eIF2B/eIF2 interactions and reduced recycling of eIF2 into its active form (eIF2-GTP), which would dampen translation initiation (13). Conversely, in agreement with our previous report (22), 4E-BP is phosphorylated in resting EOS (Table 1). This indicates that 4E-BP does not act as a blocker of eIF4E binding to the 5′ cap in resting EOS and, therefore, other factors are responsible for restricting protein translation in resting EOS. Thus, the combined lack of eIF2B phosphorylation with the phosphorylation of eEF2 on Thr56 suggests attenuation of both initiation and elongation of protein translation in resting EOS (22).

**Table 1:** Proteins involved in initiation, elongation, and termination, and present in fresh human blood EOS.

Protein/gene name	mRNA expression (RPKM)	Protein expression (iBAQ/10000)	Protein phosphorylated sites	Functional consequence of the phosphorylation state
**Initiation factors**				
EIF4E	4	28085	Not detected	eIF4E is functional?
EIF4EBP1 (4E-BP)	17	21457	T68	Allows eIF4E activity to initiate translation (9)
EIF4EBP2	46	65650	Not detected	
EIF4G1	19	12611	S1238, T1218, S1194	
EIF4G2	220	23543	Not detected	
EIF4A1	115	127510	Not detected	
EIF4B	42	37156	Y233, S406, S359, S459	
PABPC1	230	90073	Not detected	
EIF3A	20	19128	T574	
EIF5B	9	7267	S164	
EIF2A	Not detect.	7911	Not detected	
EIF2B1	22	17511	Not detected	No eIF2B/eIF2 interaction, *translation initiation is impaired* (13, 23)
CSNK2A1 (CK2)	20	24012		
CSNK2B (CK2)	7	27489		
**Elongation factors**				
EEF1A1	122	779580	Not detect.	
EEF1B2	10	170360	Not detect.	
EEF2	78	306930	T57 (Thr56), T59	Inhibits Ribosome binding, *elongation is impaired* (16)
EEF2K	2	2726	Not detected	
**Termination factors**				
ETF1	33	5269	Not detected	
GSPT1	7	42129	Not detected	
GSPT2	Not detected	204	Not detected	

*For RNA Seq analysis, reads per kilobase per million mapped reads (RPKM) > 2.0 were used as positive mRNA expression by freshly purified blood EOS (5 × 10^6^ cells) (21)*.

*75 million EOS from 3 different donors were analyzed using two-dimensional liquid chromatography coupled with high-resolution mass spectrometry to generate a proteome and phospho-proteome (3). Protein intensity-based absolute quantification (iBAQ) and phosphorylated sites are shown*.

IL-5, GM-CSF, and IL-3 are critical cytokines for EOS development and function. Each interacts with a specific α-chain receptor and a common, associated β-chain (24, 25). Not surprisingly, these receptors can generate both common and unique signals (22, 26–28). As indicated above, we have shown that 4E-BP is highly phosphorylated in resting EOS (22). After activation with IL-3, IL-5, or GM-CSF, 4E-BP phosphorylation state remains largely unaffected (22), suggesting that the increased translation induced by these cytokines is likely 4E-BP-independent. In addition to 4E-BP, we have unpublished observations indicating that EIF4B phosphorylation at Ser422 was unaffected by GM-CSF. Therefore, as for 4E-BP, eIF4B activity cannot explain the significant enhancement of translation in GM-CSF-activated cells (22). However, a slight but significant increase in the phosphorylation of eIF4B was observed in EOS activated by IL-3 for 20 h (unpublished data). This phosphorylation on Ser422 may account for the differences in translation seen in IL-3- versus GM-CSF-activated EOS (22). The signaling accounting for regulated translation in IL-3 or GM-CSF-activated EOS remains largely unstudied.

## Signaling and Protein Translation

Two major intracellular signaling pathways regulate translation in eukaryotic cells: the phosphoinositide 3-kinase (PI3K)/Akt/mTOR and the MAPK pathways. These two pathways are generally triggered by extracellular stimuli *via* membrane receptors but also respond to intracellular ATP levels and amino acid availability.

### PI3K/Akt/mTOR Signaling

Ligation of growth factors with tyrosine kinase or G-protein coupled (GPC) receptors typically leads to phosphorylation of the membrane phospholipid, phosphatidylinositol-4,5-biphosphate (PI-4,5-P_2_) into phosphatidylinositol-3,4,5-triphosphate (PIP_3_), by the class I lipid kinase PI3K. This transformation into PIP3 is reversed by phosphatases such as the phosphatase and tensin homolog deleted on chromosome 10 (PTEN) and the SH2-domain containing inositol phosphatase (SHIP) (29). PIP3 drives the phosphorylation and activation of Akt (also called, PKB), *via* 3-phosphoinositol-dependent kinase 1 (PDK1) (30). Akt activity is also augmented by the mTORC2 complex, composed of mTOR and rictor (rapamycin-insensitive companion of mTOR) (29). Akt can in turn phosphorylate and inhibit the glycogen synthase kinase 3 (GSK3) leading to dephosphorylation and activation of eIF2B with translation initiation (31). In addition, Akt phosphorylates five sites leading to the inhibition of the GTPase activity of tuberous sclerosis complex 2 (TSC2), on the small GTPase Ras homolog enriched in brain (RHEB), which in its GTP form stimulates the kinase activity of mTORC1 (32, 33). Therefore, the activity of the mTORC1 complex, composed of mTOR, RHEB, the mTOR associated protein, LST8 (MLST8), and the regulatory-associated protein of mTOR (Raptor), is downregulated by unphosphorylated TSC2 that is derepressed by Akt kinase activity.

Downstream mTORC1, the TOS (target of rapamycin signaling)-containing 4E-BP and p70S6K are phosphorylated. As seen above, phosphorylated 4E-BP is inactive and allows eIF4E to bind eIF4G to initiate translation. In dividing cells, mTOR phosphorylates p70S6K at Thr389, which in turn can phosphorylate ribosomal S6 protein (RPS6), eIF4B and programmed cell death 4 (PDCD4). While the function of phosphorylated RPS6 remains largely unknown, eIF4B and PDCD4 are positive and negative regulators, respectively, of the RNA helicase, eIF4A (34). mTORC1 also downregulates the activity of the eEF2 Kinase, which then subsequently enhances the elongation step of translation by eEF2. The general protein translational capacity is also enhanced by mTOR via increased transcription (more mRNA), and stimulation of the translation of mRNAs containing a string of 5′–pyrimidines (5′TOP mRNA) (35). In addition to its activation by growth factors, mTOR also senses cellular nutrient, oxygen, and energy level (36). As its name implies, most of mTOR effects are neutralized by rapamycin. The FKBP12–Rapamycin complex quickly binds close to the kinase domain (37), leading to mTOR conformational changes, dissociation from Raptor (38, 39) and inhibition of some of mTORC1 functions (40). By binding newly produced mTOR, FKBP12–rapamycin complex also inhibits the assembly of mTORC2 (41). Rapamycin also inhibits the binding of phosphatidic acid (PA) to mTOR, reducing the stabilization of the mTORC1 and mTORC2 complexes (42). PA is synthetized during membrane phospholipid biogenesis (43), and its intracellular level modulates the amount of rapamycin required to inhibit mTOR (44). Interestingly, low doses of rapamycin inhibit mTOR-induced p70S6K phosphorylation while much higher doses are required to block mTOR-induced 4E-BP Thr37/Thr46 phosphorylation (45). As a result, other compounds that are stronger inhibitors of mTORC1 and C2 than rapamycin, such as PP242 and AZD8055, were developed.

### mTOR Signaling in EOS

Surprisingly, mTOR has not been studied in EOS, although its inhibitor, rapamycin has shown effects on EOS *in vitro* and *in vivo*. As shown in Table 2, resting human blood EOS express relatively little mTOR, but very high amount of FKBP12. FKBP12 is bound by both rapamycin and FK506 and is required for these drugs to exert their inhibitory effects in cells. Interestingly, nanomolar doses of FK506 strongly inhibit calcium ionophore-induced cytokine (GM-CSF) production in EOS, while micromolar doses of rapamycin does not (46). Due to the competition between rapamycin and FK506 on FKBP12, high amount of rapamycin antagonizes the FK506-mediated inhibition of cytokine production in EOS (46). However, rapamycin is more potent than FK506 in inhibiting IL-5-induced prolonged EOS survival (46). The divergence between FK506 and rapamycin has also been described in T lymphocyte and mast cells, where rapamycin modulates proliferation rather that gene expression (47, 48). Another study (49) confirmed that rapamycin reduces IL-5-induced pro-survival signaling in EOS but the effect was modest and required high doses of drug for at least 72 h. In the same study, rapamycin also partially inhibited IL-5-induced eosinophil cationic protein (ECP) release from EOS (49). In addition, mTOR has important functions during EOS differentiation as rapamycin inhibited mouse EOS differentiation downstream of IL-5 (50). This is in agreement with the dependence of T cell proliferation and differentiation on mTOR (51). Remarkably, rapamycin has no inhibitory effect on mouse EOS recruitment into the BALF after exposure to dust-mite allergen in chronic allergic models (52), suggesting that the role of mTOR signaling is confined to development and possibly survival but not cell migration.

**Table 2:** Proteins present in human blood eosinophil (EOS) and involved in the phosphoinositide 3-kinase/mammalian target of rapamycin pathway.

Protein/gene name	Protein expression (iBAQ/10000)	Phosphorylated sites
AKT1 (PKB)	6656	Not detected
AKT2 (PKB)	2282	S478
FKBP1A (FKBP12)	1997100	Not detected
GSK3A	9069	Not detected
GSK3B	12297	S9
INPP5D (SHIP)	53856	S243, S971, S1039
MLST8	3323	Not detected
MTOR	1503	Not detected
PDCD4	41522	T90, S94
PDPK1	21325	Not detected
PIK3CA	94	Not detected
PIK3CB	2190	Not detected
PIK3CD	8867	Not detected
PIK3CG	9815	Not detected
PTEN	9587	Not detected
RHEB	10969	Not detected
RICTOR	1251	Not detected
RPS6	143160	S235, S326
RPS6KB1 (p70S6K)	970	Not detected
RPS6KB2 (p70S6Kb)	1869	Not detected
RPTOR	710	S863
TSC1	3037	Not detected
TSC2	2707	S1420

*75 million EOS from 3 different donors were analyzed using two-dimensional liquid chromatography coupled with high-resolution mass spectrometry to generate a proteome and phospho-proteome in resting EOS (3). Intensity-based absolute quantification (iBAQ) and phosphosites are shown*.

In both human and mouse EOS, PI3K is required for a variety of functions. These include chemokine-induced EOS granule proteins release (53), platelet-activating factor (PAF)-induced chemotaxis but not LTC4 release (54). The PI3K/Akt pathway is also essential for IL-5-induced β2-integrin adhesion to bovine serum albumin (BSA) (55), and IL-5-induced guinea pig EOS mobilization from the bone marrow (56). In EOS, the PI3K/Akt pathway can be activated by fMLP or RANTES after priming with IL-5 or IL-3 (57). Prostaglandin E_2_ (PGE_2_) *via* EP4 induces PI3K/PDK1-dependent increase in Akt phosphorylation, which consequently inhibits eotaxin-induced EOS shape changes and chemotaxis (30). Therefore, the PI3K/PDK1/Akt pathway is important in EOS and regulates a variety of functions depending on its activator.

### MAPK Signaling

The MAPK (ERK and p38) signaling pathways are involved in most of cellular functions, including differentiation and proliferation. ERK1, ERK2, p38α, and p38β are coded by four different genes (MAPK3, MAPK1, MAPK14, and MAPK11). Following intracellular or extracellular activation, the MAP kinase kinase kinases (MEKK) are activated, leading to phosphorylation of MAP kinase kinases (MEK) and, finally, MAPK are phosphorylated (58). ERK1/2 alone possess more than 150 substrates involved in a large variety of cell functions, including transcription, cell death, autophagy metabolism, and translation (59). Among the kinases activated by ERK or p38 are kinases involved in protein translation, including p90S6K (RSK), the MAPK-interacting kinases (Mnk), and the MAPK-activated protein kinase 2 (MK2) (2). The latter has an important role in 3′UTR directed, mRNA binding protein-dependent translation. P90S6K are activated by ERK signaling that can then phosphorylate TSC2 at Ser1798, activating mTORC1 and protein synthesis (60, 61). Of note, ERK may also directly phosphorylate and inhibit TSC2, leading to increased mTORC1 activity (62). Like p70S6K, p90S6K also phosphorylates both eIF4B and eEF2 kinase, which enhances eIF4B/eIF3 interactions and eEF2 function and, consequently, protein initiation and elongation (12, 23). While Mnk2 activity is thought to be constitutive, Mnk1 phosphorylation and activation can be triggered downstream ERK and p38 leading to eIF4E phosphorylation at Ser209 (63). Although this phosphorylation inhibits eIF4E binding to the 5′-cap, it may also control the translation of specific mRNAs (63).

### MAPK Signaling in EOS

Mitogen-activated protein kinases have important roles in many critical events, including EOS survival, migration, adhesion, production of inflammatory mediators, and degranulation. In EOS, ERK and p38 are phosphorylated and active following stimulation with a variety of mediators, including the β-chain cytokines (IL-3, IL-5, and GM-CSF), chemokines, fMLP, the PAF, and matrix proteins (26, 28, 53, 57, 64–71). Table 3 shows the expression levels of ERK, and their downstream targets, RSK1–3 (p90S6K), all of which are phosphorylated at a detectable level in resting cells. However, EOS contain little Mnk1/2 (Table 3), suggesting that the MAPK activation likely does not regulate protein translation *via* eIF4E phosphorylation (Figure 2); and despite its phosphorylation, the low level of Mnk2 probably have little effect on eIF4E phosphorylation. Consistent with MAPK activation, upstream MEK and MEKK were also phosphorylated at multiple sites in circulating EOS (Table 3). These data suggest that such cells are not truly resting but have been partially activated or primed either *in vivo* or during isolation.

**Table 3:** Proteins present in human eosinophil and involved in the mitogen-activated protein kinase signaling upstream of protein translation.

Protein/gene name	Protein expression (iBAQ/10000)	Phosphorylated sites
MAPK3 (ERK1)	57843	Not detected
MAPK1 (ERK2)	119320	Not detected
MAPK14 (p38α)	22843	Not detected
MAPK11 (p38β)	Not detected	Not detected
RPS6KA1 (p90S6K, RSK1)	75911	T393, S389, S372
RPS6KA2 (p90S6K, RSK3)	21015	T595, S402
RPS6KA3 (p90S6K, RSK2)	26924	T577, S227, S386, T231, S369
MKNK1 (Mnk1)	3601	S221
MKNK2 (Mnk2)	112	Not detected
MAPKAPK2 (MK2)	44194	Not detected
MAP2K2 (MEK2, upstream ERK)	153200	S226, T394
MAP2K4 (MEK4, upstream p38)	14813	S91, T89
MAP3K3 (MEKK3, upstream ERK)	5375	S178, S270, S281

*75 million EOS from 3 different donors were analyzed using two-dimensional liquid chromatography coupled with high-resolution mass spectrometry to generate a proteome and phospho-proteome in resting EOS (3). Intensity-based absolute quantification (iBAQ) and phosphosites are shown*.

## Messenger RNA-Specific Protein Translation

mRNA translation is clearly not an all or nothing event. Agonists may increase or decrease ribosomal mobilization of all, the majority or subsets of mRNAs. This may occur through a slowdown of global elongation by phosphorylated eEF2 allowing poorly translated mRNAs to enter initiation and to be translated when elongation becomes derepressed (18). Alternatively, increased eIF4A helicase activity may preferentially facilitate the translation of mRNAs possessing secondary structures in their 5′-UTR that require unwinding prior to initiation.

Selective regulation requires the recognition of unique cis-elements within the mRNA by sequence-specific mRNA binding proteins. In this way, subsets of mRNAs can be selectively identified and regulated for differential translation and mRNA decay. One well-studied example is the pyrimidine-rich domain termed terminal oligopyrimidine (TOP). mRNA containing TOP usually code for elongation factors and ribosomal proteins (72) and their translation is preferably induced by the mTOR pathway (73). We will discuss additional examples below.

### IL-3 Induces Translation of Semaphorin-7A mRNA in EOS

Semaphorin-7A mRNA level is relatively high in resting cells and changes only slightly in activated blood EOS. However, its translation remains almost undetectable despite GM-CSF activation (22). Surprisingly, despite similar mRNA levels, the translation rate for semaphorin-7A is more than 10-fold higher in IL-3- versus GM-CSF-activated EOS (22). Consistent with increased translation, semaphorin-7A mRNA was enriched in polyribosome fractions following IL-3 compared to GM-CSF (22). Of note, TOP mRNAs (EEF1A1 and PABP) were not enriched in the polyribosome fraction by IL-3, suggesting unique and highly selective signaling from IL-3 receptor to the translational machinery.

Freshly purified blood EOSs possess surface semaphorin-7A, which tends to decrease overtime during the first 20 h of cell culture (unpublished data). Activation with IL-5 or GM-CSF maintains or slightly increases surface semaphorin-7A over this same time span (27). On the other hand, over a broad range of doses, IL-3 significantly increased surface semaphorin-7A expression (27). Interestingly, IL-3-induced semaphorin-7A translation occurred more than 6 h after activation (unpublished data), suggesting that considerable signaling and possibly the translation of accessory proteins precedes semaphorin-7A translation initiation.

### ERK/p90S6K/RPS6 Signaling Downstream from the β-Chain Cytokines in EOS

Along with RL13A (74), RPS6 is one of the rare ribosomal proteins that is phosphorylated following cellular stimulation in eukaryotic cells (75, 76). In stromal cells, RPS6 phosphorylation is directly controlled by the kinases p70S6K1 and p70S6K2, downstream of mTOR (77). In knock-in mice, genetically modified at RPS6 phospho-sites, aggregate protein synthesis was decreased in liver and embryonic fibroblasts (78). Other studies have suggested that RPS6 phosphorylation facilitated more efficient 40S ribosomal subunit assembly (79). This idea is supported by structural and biochemical data demonstrating that phosphorylated RPS6 is located at the interface between the small and the large ribosomal subunits near the tRNA-binding sites (80), and is enriched in polyribosomes (75). The correlation of RPS6 phosphorylation with cell division during mitogenic activation suggests that RPS6 participates in translation control in dividing cells (81). However, the role of phosphorylated RPS6 in non-dividing cells, such as EOS, remains unexplored.

We found that all β-chain cytokines strongly induced RPS6 phosphorylation at Ser235 and Ser236. However, while RPS6 phosphorylation persisted for only 1–4 h in EOS culture with IL-5 or GM-CSF, IL-3 induced continuous RPS6 phosphorylation for as long as IL-3 remained present in the culture medium (22). Of note, this unique feature of IL-3 to prolong RPS6 phosphorylation has also been observed in basophils (82, 83). Anti-IL-3 neutralization rapidly reversed RPS6 phosphorylation indicating that constant presence of IL-3 was required and that signaling was likely driven by a labile secondary messenger following IL-3 activation (22). Interestingly, the relatively rapid RPS6 dephosphorylation in GM-CSF-activated EOS was phosphatase 1 (PP1)-dependent, although total PP1 activity in cell lysates was the same in GM-CSF- and IL-3-activated EOS (22). This suggests that PP1 activity toward RPS6 may be negatively regulated only in IL-3-activated but not in GM-CSF-activated EOS. Of note, a 23 KDa ribosomal-associated inhibitor of PP1, termed ribosomal-associated inhibitor of phosphatase 1 (RIPP1) has been identified but remains incompletely described (84, 85).

As mentioned above, RPS6 can be phosphorylated downstream of the PI3K/Akt/mTOR/p70S6K pathway (77). However, in EOS neither rapamycin, PI3K nor p70S6K inhibitors prevented IL-3-induced RPS6 phosphorylation (22). On the contrary, p90S6K (RSK) inhibitors significantly reduced IL-3-induced, RPS6 phosphorylation on both Ser235 and Ser236 (22). GM-CSF activation of p90S6K peaked after 10 min, and p90S6K was already largely dephosphorylated by 1 h (22). Conversely, progressive phosphorylation of p90S6K occurred after IL-3, peaking, at 16–20 h and still detectable until IL-3 was removed or neutralized in the culture medium (22). P90S6K was the first RPS6-phosphorylating kinase described in *Xenopus* oocytes (86), but has since been implicated in cell proliferation and survival (87). P90S6K includes three isoforms (RSK1, 2, and 3), all with inducible phosphorylation-dependent activity and similar functions. P90S6K phosphorylation is downstream of ERK and phosphorylated p90S6K has been found associated with polyribosomes (88). Phosphorylation on Thr573 is sequentially followed by Thr359, Ser363, and finally Ser380. All four sites are strongly phosphorylated following IL-3-activated EOS (22). Ultimately 3′–phosphoinositol-dependent kinase-1 (PDK1) phosphorylates Ser221 leading to maximal p90S6K activation (89). In addition to RPS6, p90S6K also phosphorylates eIF4B and GSK3 (12, 90). Phosphorylated eIF4B interacts with eIF3A, enhancing translation initiation (91). P90S6K inactivates GSK3, which would in turn dephosphorylate and activate eIF2B, thus promoting eIF2 recycling and increasing translation initiation [(90); Figure 2]. While the dephosphorylation of eIF2B possibly occurs *via* changes in PP1 activity (90), differential activation of p90S6K by the different β-chain cytokines was not accompanied by changes in PP1 activity (22), suggesting that IL-3-induced and prolonged p90S6K activation does not affect translation *via* the GSK3/PP1/eIF2B pathway. As proposed above, the β-chain cytokines could differentially regulate a ribosomal specific PP1 regulatory protein (85).

Upstream, p90S6K phosphorylation is known to be regulated by the MAPK and particularly by ERK1/2 (92). Consistent with these data, a selective inhibitor of both MEK1 and MEK2 (U0126) added 3 h after IL-3, blocked the phosphorylation of p90S6K on Ser380 and RPS6 in EOS in culture. Another MAPK, p38, has also been implicated as a potential activator of p90S6K in dendritic cells (93). However, a p38 inhibitor (SB203580) had no effect on p90S6K phosphorylation in IL-3-activated EOS.

In addition to semaphorin-7A, we have more recently shown that the low-affinity IgG receptors, FCGR2B and FCGR2C (CD32B and CD32C) were upregulated at the translational level by IL-3, in a p90S6K-dependent manner (94). Therefore, we have so far identified two transcripts whose translation is exclusively enhanced by the prolonged effect of IL-3 through ERK/p90S6K signaling. MS proteomic analysis of EOS treated with IL-3 with and without ERK inhibitors will yield insight into the identity of other similarly regulated mRNA.

## mRNA-Binding Proteins and Control of Protein Translation

### Overview

RNA-binding proteins (RBP) regulate all aspects of RNA metabolism, including biogenesis, cellular localization and transport, stability, and translation. With the emergence of high throughput screening and quantitative proteomics, several hundred (approximately 500) potential RBP have been identified (95). Given their obvious importance, enormous effort has been directed to expand our knowledge on how RNA-protein interactions determine RNA function and cell fate. It bears reiterating that mRNA is not a rod but a complex 3-dimensional shape. As such, RBP can interact with mRNA *via* structure, sequence or structure, and sequence elements. A simple example is 5′-cap binding protein eIF4E. A more complex example is PABP, which interacts with poly A tails, a combination of sequence (Poly A) and structure. The iron-response binding protein (IRE-BP) interacts with a sequence presented on a stem-loop and bulge (96). Alterations in the size of the loop, the distance between the loop and bulge or the loop sequence ablates binding. Given these levels of target specificity, some RBPs will no doubt be successfully targeted with therapeutics to treat human disease.

Once transcribed from genomic DNA, newly produced pre-mRNAs are immediately covered by a number of nuclear RBP to protect from degradation by nucleases, guide splicing and prepare for cytoplasmic transport. As mature mRNA are translocated, the inventory of bound proteins are often replaced with a new set of RBP that determine intracellular location, define degradation rates as well as translatability (see above) in the cytoplasm. In response to extrinsic and intrinsic stimuli, free and bound RBP are subject to post-translational modifications (PTM) (e.g., phosphorylation, ubiquitination, acetylation, and methylation) that may induce conformational changes and alter the association between RBP and target mRNA (97, 98). Depending on stimulus and cell type, the modified RBP may associate with or dissociate from mRNA, affecting the transcript stability as well as its translation, clearly affecting protein production. RBP bind to RNA *via* a variety of domains, including the so-called RNA-recognition motif (RRM), zinc finger motives, K-homology domains (KH), RGG boxes, and DEAD/DEAH boxes (97). Often more than one binding domain are present allowing simultaneous interactions with multiple mRNAs, multiple sites within one mRNA target or between specific mRNA sequences and organelles such as ribosomes or stress granules. RBP can also form higher order structures through protein–protein interactions either as homo- or heterodimers/trimers, etc. As a rule, RBP that interact with 5′ or 3′ ends of mRNA often regulate translation initiation (e.g., translation initiation factors and their partners; PABP) while those that bind to coding regions can affect translation, localization, or mRNA decay (e.g., IRE-BP). 3′ UTR RBP (e.g., AUF1, HuR, TTP, TIA-1, TIAR, FMRP, PTB, KSRP, hnRNPs, nucleolin, and CUGBP) are most often involved in mRNA localization and decay (99).

### Regulation of mRNA Binding Proteins in EOS and Their Potential Effect on Protein Translation

It is well known that many rapidly inducible mRNA coding for pro-inflammatory cytokines and oncoproteins are very short-lived. Inevitably, these mRNA contain *cis*-acting sequences into their 3′-UTR (100). The best-characterized instability determinant is composed of adenosine-uridine (AU)-rich element (ARE) repeats that are found in 3′-UTR of GM-CSF, IL-3, IL-5, IL-2, IFN-γ, and TNF-α and other cytokine mRNA. The life-span of ARE mRNA are regulated by a subset of binding proteins (AUBPs) that preferentially target the ARE and stabilize or further destabilize the transcripts. To date, approximately 20 AUBP have been identified. EOS express 7 AUBP (AUF1, hnRNP C, YB-1, nucleolin, TIA-1, HuR, and BRF1) (3) and their role in the regulation of mRNA stability has been demonstrated by many studies (101–105). In response to an exogenous pro-survival signal, Y-box binding protein 1 (YB1) and heterogeneous nuclear ribonucleoprotein C (hnRNP C) became associated with, while heterogeneous nuclear ribonucleoprotein D (hnRNP D or AUF1) dissociated from the ARE of GM-CSF mRNA (101, 106). These interactions were accompanied by the multiple phosphorylation of AUF1 (Ser83, Ser87, and Thr91) likely by ERK, CK1, GSK3β, or PKA (103, 107–109). Presumably, phosphorylation reduced the affinity of AUF1 for the ARE. AUF1 also undergoes post-transcriptional, alternative splicing events (110), yielding four AUF1 mRNAs and isoform variants (p37, p40, p42, and p45), all of which are detectable in human EOS (106). Thus, the regulatory control by AUF1 isoforms appears to be highly complex and includes their potential to form heterodimers (111) with a different affinity for ARE containing mRNAs (p37 > p42 > p45 > p40) (112, 113). While AUF1 has additional functions, its best-characterized function is to accelerate the decay of associated ARE-rich mRNAs. The p37 isoform has been shown to interact with the exosome in EOS (103) and exhibit the greatest destabilizing activity toward ARE-containing mRNAs compared to other isoforms (114).

mRNA turnover is often linked to translation (115) and the role of AUBP in RNA translation has been extensively studied in many systems. Similar mechanisms may occur in EOS although no direct evidence has yet been published. As mentioned above, PI3K/Akt/mTOR and MAPK cascades are the major signaling pathways that control global RNA translation upstream of the ribosomal machinery. These pathways have also been linked to AUBP-mediated mRNA decay in many cell types. EOS possess all translational machinery (Table 1) and can activate those kinase pathways when stimulated with various agonists (fMLP, RANTES, eotaxin, IL-5, IL-3, and PGE2) (30, 57). For example, ERK is activated by hyaluronic acid, IL-3 or IL-5, and likely drives AUF1 phosphorylation (102, 103), impacting the translation and decay of multiple ARE mRNA, including GM-CSF. These data suggest that ARE mRNA will be subject to translation control in EOS. This has been investigated by analysis of transfected mRNA, which has revealed striking differences in protein production despite similar cytosolic steady state levels of coding mRNA (116). Below, we discuss well-defined AUBP and their potential roles in target mRNA translation in EOS.

#### Heterogeneous Nuclear Ribonucleoprotein D

In eukaryotic cells, AUF1 (hnRNP D) is one of the best-characterized AUBPs and has a multiplicity of functions. It is a positive regulator of mRNA translation (117) but can also accelerate transcript decay. These two events may or may not be coupled. For example, AUF1 weakly targets Myc mRNAs for an accelerated decay but strongly promotes its translation by successfully competing with the cytotoxic granule-associated RNA binding protein TIA-1 and TIA-1-like 1 (TIAR) for a common binding site (118). Consistent with this observation, cells lacking AUF1 exhibited an increase binding of the translation-inhibitory TIA-1/TIAR to ARE mRNA, resulting in translation repression of the mRNA encoding TGF-β-activated kinase 1 (TAK1) and IL-10 (119, 120). Depending on the cell and its activation state, AUF1 can also assemble factors necessary for mRNA translation, including eIF4G, chaperones (hsp27 and hsp70), and PABP, thereby affecting translation (121–123). In EOS, eIF4G is phosphorylated by a brief (5 min) exposure to IL-5 (3), a condition that favors AUF1 phosphorylation and global protein translation (22). As EOS express high levels of PABP-C1 (major cytoplasmic PABP isoform) (3, 124), activated AUF1 may facilitate the displacement of TIA-1/TIAR by PABP-C1 and promote phospho-eIF4G-mediated translation initiation. Taken together, these results strongly indicate that modulation of translation efficiency by AUF1 is a common cellular event, which may not necessarily couple with ARE-mediated decay. Interestingly, AUF1 can also function as an inhibitor as was reported in EV71 virus translation. In this model, AUF1 binding to a stem–loop structure within IRES displaced HuR and Ago2, whose association promotes IRES-dependent translation and subsequent viral replication.

#### Y-Box Binding Protein-1

In EOS, an increase in YB-1 content led to the stabilization of GM-CSF mRNA. Binding was mediated through 3′ UTR ARE and resulted in increased GM-CSF translation and release with subsequent pro-survival signaling (101, 102). YB-1 can also stabilize non-ARE containing mRNA (125, 126), suggesting that it associates with other cis-elements or acts through another protein effector(s). Consistent with this notion, as the YB-1/mRNA ratio increases, so does the translation efficiency of the affected mRNA (125, 126). At high YB-1/mRNA ratios associated with maximal mRNA stabilization, YB-1 displaces eIF4F from the messenger ribonucleoprotein (mRNP) complex, possibly inhibiting the translation of the stabilized mRNA (127). This mechanism was not observed in EOS for GM-CSF expression, however (101, 102), which may reflect the ordinarily high basal levels of YB-1 in these cells. Thus, it is not entirely clear how endogenous AUBP such as YB-1 influence eIF4F-mRNA interactions and regulate mRNA stability and translation in these cells. In cells, at low YB-1/mRNA ratios, eIF4F is known to bind effectively to mRNA near the 5′ cap-structure and drive translation (125, 126). YB-1 can be phosphorylated at a single site (Ser316) within the C-terminal domain (CTD) by multiple kinases (Akt, ERK2, GSK3-β, and JNK) (3), which leads to increased IL-2 mRNA stability and cytokine production (128). YB-1 binds to mRNA as a monomer through the cold-shock domain (CSD) and the CTD (125), which can unfold mRNA secondary structures, likely facilitating interactions with the translation initiation machinery. Inhibition of translation is mainly attributed to the CTD. Similarly to the full length YB-1, CTD displaces eIF4G from mRNP while the CSD displaces eIF4E, eIF4A, and eIF4B by interacting with the 5′-Cap-structure or with its adjacent region (125, 126). After EOS exposure to IL-5, eIF4G (Ser1238, Thr1218, and Ser1194) and eIF4B (Tyr233, Ser406, Ser359, and Ser459) are rapidly phosphorylated (3). YB-1 can also be phosphorylated by Akt (98, 129, 130), which lowers its affinity for the 5′-cap-structure (or/and adjacent mRNA region) (130). This may also facilitate the assembly of the translation initiation complex. Of note, circadian changes of YB-1 binding to GM-CSF mRNA have been observed in circulating EOS from subjects with nocturnal asthma, with lower YB-1/GM-CSF mRNA interaction at 04.00 a.m., suggesting possible increased GM-CSF protein production and EOS activation at night (131).

#### Heterogeneous Nuclear Ribonucleoprotein C

Heterogeneous nuclear ribonucleoprotein C has been predominantly associated with the regulation of mRNA stability although several reports describe translational regulation through 5′ UTR interactions (132–135). This function was first identified in rabbit reticulocyte lysate supplemented with exogenous hnRNP C. Those studies revealed hnRNP C bound to a non-ARE domain, stabilizing APP mRNA and increasing its translation (132). In neurons, hnRNP C and FMRP were shown to compete for binding to a coding region element of APP mRNA that modulated APP mRNA translation in opposite directions (136). Further study clarified that increased APP translation by hnRNP C was accompanied by enhanced mRNA polyadenylation, which was mediated by a functional IRES found in the 5′ UTR of the transcript (137). Thus, the mRNA-specific translational activation by hnRNP C is generally independent of ARE and is through interactions with distinct 3′ or coding region (132, 136) target sequences, IRES (133), 5′ UTR, or heptameric U sequence in IRES (138). Whether similar mechanisms occur in EOS is unknown although hnRNP C was reported to bind GM-CSF mRNA and associated with transcript stability (103). To date, neither cytoplasmic kinases nor phosphosites on hnRNP C have been identified although several RNA-dependent protein kinases (PKA, PKC, CDK-II, and PKR) have been associated with hyperphosphorylation of hnRNP C1 (small isoform of hnRNP C) in nuclear extracts (139).

#### Other AUBPs

HuR (stabilizer of ARE mRNA) and TIA-1 (U-rich binding protein) bind to GM-CSF and TGF-β mRNA and are associated with transcript stability in EOS. While the role of HuR in mRNA translation has not been reported, TIA-1 is believed to repress the translation of TNF-α (140), COX-2 (141), cytochrome c (142), and 5′ TOP mRNAs (143). TIA-1 binds to the ARE of TNF-α mRNA, but has no effect on the mRNA decay. Instead, TIA-1 represses TNF-α translation by promoting its sequestration in non-polysomal mRNP complexes or the so-called stress granules (144). TIA-1 can also recruit multifunctional RBP, including PTB, La, hnRNP K, and hnRNP A1, all of which are expressed by EOS (145). However, it remains unknown whether this recruitment is associated with TIA-1-mediated translational repression. TIA-1 can be phosphorylated by FASTK but the phosphorylation sites have not been mapped (146, 147). On a similar note, the mRNA stabilizing protein, Sjögren syndrome type B antigen (SSB or La) plays a unique role in translation initiation (148–151). La is largely nuclear but acts as an RNA chaperone in the cytoplasm when translation starts. La binds in close proximity to the translation start site and unwinds second structure of mRNA to expose embedded AUG start codons. Similar actions were also observed for the translation of virus-encoded mRNA (152–154). This unique feature of La is critically important in facilitating translation initiation because the translation start sites of certain mRNA are buried in strong stem–loop or secondary structures and are not efficiently recognized by the scanning 43S ribosomal subunit. La is phosphorylated on Thr301, Ser366, and Thr389 by AKT and CK2 (151, 155, 156), which contributes to its nuclear or cytoplasmic distribution (157).

## Regulation of Translation and Potential Therapeutic Targets

### Endogenous GM-CSF Effects on EOS Biology and the Use of Pin-1 As a Potential Therapeutic Target

We have described above how RBP regulate mRNA stability and translatability, particularly of GM-CSF mRNA in EOS. GM-CSF plays a pivotal role in the modulation of EOS differentiation, function, and survival. The cytokine is upregulated in eosinophilic diseases and a major contributor to enhancing EOS survival in the lungs of patients during active asthma (158). Recombinant GM-CSF promotes EOS survival about five times as potently as equal concentrations of IL-5 (159). In asthmatics, GM-CSF is produced by a wide spectrum of cell types, including lung epithelial cells, lymphocytes, alveolar macrophages, EOS, endothelial cells, and fibroblasts. As EOS typically increase by 20-fold in the lung within a few days of an allergen challenge (160), autocrine GM-CSF is an important source in order to support survival. The level of endogenous GM-CSF in BAL fluid is low (161–163) compared to IL-5 in both mice and humans (164). However, intranasal delivery of Adeno-GM-CSF to the airways of OVA-sensitized mice resulted in sustained accumulation of various inflammatory cell types, most noticeably EOS, in the lung for more than 2 weeks post OVA aerosol challenge (165, 166). Conversely, neutralization of endogenous GM-CSF during aeroallergen exposure significantly inhibited eosinophilic inflammation and airway hyper-responsiveness. This suggests that small amount of endogenous GM-CSF can significantly contribute to the development and persistence of eosinophilic airway inflammation. *In vitro*, purified peripheral blood EOS synthesize small amounts (~1 pg/ml) of anti-apoptotic GM-CSF (167, 168), after stimulation with a variety of factors (fibronectin, hyaluronic acid, TNF-α, IL-3, IL-5, IL-15, integrins, IFN-γ, calcium ionophore, cross-linking of cell surface molecules) (67, 102, 167, 169–174). Activation-induced survival was blocked by the addition of neutralizing anti-GM-CSF even 2 days after the initiation of culture, indicating that the cells continuously release low levels of GM-CSF on which survival depends (103, 172–174). Similarly, the majority of BAL EOS obtained 48 h after segmental allergen challenge died *in vitro* at 6 days in the presence of neutralizing anti-GM-CSF. Both *in situ* hybridization (tissue EOS) (175) and qPCR (purified EOS) (67, 172, 173) analyses have demonstrated that increased GM-CSF mRNA was associated with GM-CSF protein secretion and prolonged EOS survival.

#### Pin1

While all ARE mRNA have relatively short half-lives (20 min-2 h), GM-CSF mRNA is extremely labile (*t*1/2 < 6 min) in resting EOS but show significantly increased stability (increased by fourfold to sixfold) after cell activation. This conversion likely reflects alterations in the composition of interacting AUBP (67, 103, 172, 173). Multiple AUBP, including AUF1, HuR, YB-1, and hnRNP C associate with and regulate the decay of GM-CSF mRNA in EOS (101–103). Cell activation triggered occupancy of GM-CSF mRNA by YB1, hnRNP C, and HuR, which displaced AUF1. ERK-mediated phosphorylation likely caused a decrease in affinity for GM-CSF mRNA by AUF1 (103), which led to remodeling of the GM-CSF mRNP complex. Co-immunoprecipitation and gene knockout studies have found that Pin1, a *cis-trans* peptidyl prolyl isomerase, interacts with multiple AUBPs, including AUF1, HuR, KSRP, SLBP, and the translation regulators eIF4E and 4E-BP1/2 (103, 176–178). Pin1 is essential for cell-cycle progression through interactions with cyclinD (179). Pin1 is the only known eukaryotic isomerase with specificity for Ser-Pro or Thr-Pro peptide bonds. Isomerization is bidirectional with *cis* to *trans* or *trans* to *cis* conversions but occurs approximately 1000-fold faster when the N-terminal Ser or Thr has been phosphorylated (180). Structurally, Pin1 has two domains, including a ~40 amino acid *N*-terminal WW domain and a C-terminal isomerase domain. The WW domain binds pSer/pThr-Pro motifs while the catalytic domain is responsible for substrate isomerization. Pin1-mediated isomerization has profound effects on target-protein folding, altering subsequent protein–protein and protein–nucleic acid interactions, protein stability and subcellular localization thereby altering a variety of cellular processes, including cell cycle progression, apoptosis, innate and acquired immunity, and gene regulation. We showed that Pin1 was reproducibly pulled down with AUF1 in human EOS and T cells irrespective of cell activation (103, 105). Cell activation also increased Pin1 activity, which likely isomerized phosphorylated AUF1. These events occurred with a simultaneous reduction of AUF1 binding to GM-CSF mRNA. Conversely, inhibition of Pin1 reduced isomerase activity, reconstituted the AUF1–GM-CSF mRNP complex and accelerated transcript decay. Consistent with this *in vitro* data, EOS obtained from the blood or BALF from patients with active asthma showed significantly elevated Pin1 isomerase activity. *In vivo* Pin1 blockade significantly reduced pulmonary EOS counts, GM-CSF production, and cell viability in rat models of asthma (181). These observations indicate that Pin1 is a critical regulator of GM-CSF mRNA turnover and production, which in turn controls the survival of activated EOS in the lungs of asthmatics.

In addition to its role in mRNA stabilization, Pin1 signaling amplifies or suppresses the action of kinases, phosphatases, transcription factors, cell cycle regulators, and apoptotic effectors (124, 180). This broad targeting specificity of Pin1 arises from its short consensus target (pSer/pThr-Pro) as well as the phosphorylation frequency of S/T-P sites, which are found in numerous proteins. Pin1 activity can be modulated either positively or negatively without change in protein content, in response to injury or environmental cues. Chronic activation or suppression can be pathologic as seen in immune disorders, fibrosis, cancer, and neurodegeneration (105, 182, 183). Specifically, Pin1 overexpression or amplification is highly correlated with cancer progression and metastasis while Pin1 loss is seen in evolving Alzheimer Disease. Pathology may result from loss of regulation of RBP with alterations in cytokine mRNA stability and translation. Thus, pharmacologic modulation of Pin1 activity with small molecule inhibitors may provide a novel approach to eosinophilic diseases, such as asthma. Unfortunately, current Pin1 inhibitors lack specificity or are excessively toxic.

### IL-3 Signaling in EOS

TPI ASM8 is a drug in development, targeting the common β-chain receptor for all IL-5, GM-CSF, and IL-3, in the form of RNA-targeted inhaled oligonucleotide antisense phosphorotioates (184). Although TPI ASM8 seems to be well tolerated and leads to some reduction of EOS and eosinophilic hematopoietic progenitor (CD34^+^IL5R^+^), other alternative therapeutic targets more specific to each of the 3 cytokine should be developed.

So far, we have identified semaphorin-7A and FCGR2B/C (CD32) as specific genes exclusively responding to IL-3 activation *via* prolonged ERK/p90S6K signaling. It is probable that additional genes are similarly regulated at a translation level by IL-3/ERK/p90S6K. Likely other candidate genes may share specific mRNA *cis*-elements whose identity may be inferred by homology searches among IL-3 upregulated mRNA. We started analyzing how semaphorin-7A or FCGR2 affect EOS function. We found that IL-3-activated EOS adhere to the only known semaphorin-7A ligand, plexin-C1 (27). Plexin-C1 is expressed by many cell types, including lymphocytes, monocytes, dendritic cells, and neutrophils (185), and has an important role in the migration of these inflammatory cells. Plexin-C1 is also expressed by stromal cells (186), which could facilitate migration or activate EOS in fibrotic tissue. Interestingly, IL-3-activated EOS migration on plexin-C1 was largely resistant to semaphorin-7A blockade while neutralizing anti-αMβ2 integrin were far more inhibitory (187). Migration in the absence of chemotaxis indicates that a haptotaxis process is operative for plexin-C1- or periostin-mediated migration (187, 188). Semphorin-7A signaling may also skew fibroblasts toward a pro-fibrotic, more mesenchymal phenotype (27, 189, 190), although we recently demonstrated anti-fibrotic functions for endogenous semaphorin-7A expressed by lung fibroblasts (191).

The upregulation of CD32 by IL-3 on EOS has a profound impact on EOS function. EOS-driven pathology in tissue requires both EOS migration from circulating blood to the site of inflammation and the release (degranulation) of preformed toxic proteins and mediators of the inflammation. IL-3-activated EOS strongly degranulate on heat-aggregated (HA)-IgG, with extrusion of ~25% of their total cellular eosinophil-derived neurotoxin (EDN) in 6 h (94) compared to less than 10% after IL-5 (94). Degranulation on HA-IgG was CD32-dependent (94). Thus, IL-3 and its downstream intracellular effectors may be potential therapeutic targets to limit EOS degranulation and EOS-driven pathologies. The use of anti-IL-5 therapies on patients with severe eosinophilic asthma has reduced asthma exacerbations and blood eosinophilia (192, 193), *see other article by Nair in this issue*. However, airway EOS are still present and active despite treatment (194, 195). This partially reflects loss of the surface IL-5 receptor expression by airway EOS (22, 196, 197). Conversely, IL-3 and the surface IL-3 receptor are upregulated and highly expressed on airway EOS (27, 198). Therefore, combined targeting of the IL-3 and IL-5 pathways may provide additive or synergistic benefits.

#### Ribosomal S6 Protein

Whether RPS6 phosphorylation in EOS induces a unique profile of proteins (e.g., semaphorin-7A, CD32, etc.), downstream of IL-3/ERK/P90S6K signaling, is unclear. If so, phospho-RPS6 would be a possible therapeutic target to reduce EOS-related pathology. On the positive side, knock-in mice lacking the ability to phosphorylate RPS6 have modest deficits (199) and show limited changes in global protein synthesis *in vivo* and in embryonic fibroblasts (78). However, β-cell development may be adversely affected by RPS6 knock-in (78). RPS6 phosphorylation can be blocked in EOS by small molecules inhibitors of p90S6K, such as BI-D1870 (200). However, the consequences of p90S6K inhibition probably include transcriptional silencing, blockade of cell proliferation, and cell death (87, 201). Thus, while potentially attractive, inhibition of this pathway remains hypothetical.

## Conclusion

The three β-chain cytokines, IL-3, IL-5, and GM-CSF are all present in human eosinophilic diseases and have both highly redundant and yet critically unique roles in the EOS biology. Their signaling affects differentiation, maturation, survival, migration, piecemeal release of immune-mediators, and degranulation. IL-3 is unique among the β-chain cytokines in generating prolonged intracellular signaling leading to the translation of a subset of EOS mRNA. Signaling requires ERK and p90S6K activation and culminates in the phosphorylation of RPS6. The control of both translation and decay of cytokine mRNA ultimately involves an interplay between mRNA-BP, especially those that target ARE. The AUBP in turn are often regulated by the action of Pin1, leading to multi-level control over cytokine gene expression. Critical, unanswered questions include the identification of RPS6-dependent mRNA as well as additional Pin1 RBP interactors and whether drugs can be developed to target these important pathways.

## Author Contributions

All authors listed have made a substantial, direct, and intellectual contribution to the work and approved it for publication.

## Conflict of Interest Statement

The authors declare no conflict of interest other than an issued patent on the use of Pin1 as a drug target to treat eosinophilia. The reviewer KWG and the handling editor declared their shared affiliation, and the handling editor states that the process nevertheless met the standards of a fair and objective review.

## Abbreviations

aa, aminoacyl; AMPK, AMP-activated protein kinase; ARE, adenosine-uridine (AU)-rich element; AUBP, ARE binding protein; AUF1 or hnRNP D, heterogeneous nuclear ribonucleoprotein D; CDK1, cyclin-dependent kinase 1; CSNK2A1, casein kinase 2; EDN, eosinophil-derived neurotoxin; eEF, eukaryotic elongation factor; eIF, eukaryotic translation initiation factor; EOS, eosinophil; FCGRII (CD32), receptor for Fc fragment of IgG, low affinity II; GEF, guanine-nucleotide-exchange factor; GTP, guanosine triphosphate; HA-IgG, heat-aggregated-IgG; hnRNP C, heterogeneous nuclear ribonucleoprotein C; La or SSB, Sjögren syndrome type B antigen; MAPK, mitogen-activated protein kinase; mRNP, messenger ribonucleoprotein; mTOR, mammalian target of rapamycin; mTORC1, rapamycin complex 1; p90S6K (RSK), 90-KDa ribosomal S6 kinase; PA, phosphatidic acid; PABP, poly-A binding protein; PIC, 43S preinitiation complex; RBP, RNA-binding protein; RIPP1, ribosomal-associated inhibitor of phosphatase 1; RPS6, ribosomal protein S6; TIA-1, cytotoxic granule-associated RNA binding protein; TIAR, cytotoxic granule-associated RNA binding protein like 1; TOP, terminal oligopyrimidine 5′-UTR, 5′ untranslated region; YB-1, Y-Box Binding Protein-1.
